# Carbon Cycling of Lake Kivu (East Africa): Net Autotrophy in the Epilimnion and Emission of CO_2_ to the Atmosphere Sustained by Geogenic Inputs

**DOI:** 10.1371/journal.pone.0109500

**Published:** 2014-10-14

**Authors:** Alberto V. Borges, Cédric Morana, Steven Bouillon, Pierre Servais, Jean-Pierre Descy, François Darchambeau

**Affiliations:** 1 Chemical Oceanography Unit, Université de Liège, Liège, Belgium; 2 Department of Earth and Environmental Sciences, KU Leuven, Leuven, Belgium; 3 Ecologie des Systèmes Aquatiques, Université Libre de Bruxelles, Bruxelles, Belgium; 4 Research Unit in Environmental and Evolutionary Biology, University of Namur, Namur, Belgium; University of Vigo, Spain

## Abstract

We report organic and inorganic carbon distributions and fluxes in a large (>2000 km^2^) oligotrophic, tropical lake (Lake Kivu, East Africa), acquired during four field surveys, that captured the seasonal variations (March 2007–mid rainy season, September 2007–late dry season, June 2008–early dry season, and April 2009–late rainy season). The partial pressure of CO_2_ (pCO_2_) in surface waters of the main basin of Lake Kivu showed modest spatial (coefficient of variation between 3% and 6%), and seasonal variations with an amplitude of 163 ppm (between 579±23 ppm on average in March 2007 and 742±28 ppm on average in September 2007). The most prominent spatial feature of the pCO_2_ distribution was the very high pCO_2_ values in Kabuno Bay (a small sub-basin with little connection to the main lake) ranging between 11213 ppm and 14213 ppm (between 18 and 26 times higher than in the main basin). Surface waters of the main basin of Lake Kivu were a net source of CO_2_ to the atmosphere at an average rate of 10.8 mmol m^−2^ d^−1^, which is lower than the global average reported for freshwater, saline, and volcanic lakes. In Kabuno Bay, the CO_2_ emission to the atmosphere was on average 500.7 mmol m^−2^ d^−1^ (∼46 times higher than in the main basin). Based on whole-lake mass balance of dissolved inorganic carbon (DIC) bulk concentrations and of its stable carbon isotope composition, we show that the epilimnion of Lake Kivu was net autotrophic. This is due to the modest river inputs of organic carbon owing to the small ratio of catchment area to lake surface area (2.15). The carbon budget implies that the CO_2_ emission to the atmosphere must be sustained by DIC inputs of geogenic origin from deep geothermal springs.

## Introduction

Freshwater ecosystems are frequently considered to be net heterotrophic, whereby the consumption of organic carbon (C) is higher than the autochthonous production of organic C, and excess organic C consumption is maintained by inputs of allochthonous organic C [1]. Net heterotrophy in freshwater ecosystems promotes the emission of carbon dioxide (CO_2_) to the atmosphere [2], [3], [4], [5], [6], [7], [9], [10], with the global emission from continental waters estimated at ∼0.75 PgC yr^−1^ [4] (0.11 PgC yr^−1^ from lakes, 0.28 PgC yr^−1^ from reservoirs, 0.23 PgC yr^−1^ from rivers, 0.12 PgC yr^−1^ from estuaries, and 0.01 PgC yr^−1^ from ground waters). More recent studies provided even higher CO_2_ emission estimates. Tranvik et al. [7] revised the CO_2_ emission from lakes to 0.53 PgC yr^−1^, while Battin et al. [6] estimated CO_2_ emission from streams at 0.32 PgC yr^−1^. Aufdenkampe et al. [8] estimated a total CO_2_ emission of 0.64 PgC yr^−1^ for lakes and reservoirs, a total of 0.56 PgC yr^−1^ for rivers and streams, and a massive 2.08 PgC yr^−1^ for wetlands. Raymond et al. [10] estimated an emission of 1.8 PgC yr^−1^ for streams and rivers and 0.32 PgC yr^−1^ for lakes and reservoirs. Such emissions of CO_2_ from continental waters exceed the net sink of C by terrestrial vegetation and soils of ∼1.3 PgC yr^−1^ [4] as well as the sink of CO_2_ in open oceans of ∼1.4 PgC yr^−1^ [11].

However, our present understanding of the role of lakes on CO_2_ emissions could be biased because most observations were obtained in temperate and boreal (humic) systems, and mostly in medium to small sized lakes, during open-water (ice-free) periods. Much less observations are available from hard-water, saline, large, or tropical lakes. Tropical freshwater environments are indeed under-sampled compared to temperate and boreal systems in terms of C dynamics in general, and specifically in terms of CO_2_ dynamics. In an extensive compilation of CO_2_ concentration data from 4902 lakes globally [12], there were only 148 data entries for tropical systems (∼3%). Yet, about 50% of freshwater and an equivalent fraction of organic C is delivered by rivers to the oceans at tropical latitudes [13]. Tropical lakes represent about 16% of the total surface of lakes [14], and Lakes Victoria, Tanganyika, and Malawi belong to the seven largest lakes by area in the world. Current estimates assume that areal CO_2_ fluxes are substantially higher in tropical systems than in temperate or boreal regions (often ascribed to higher temperatures) [8]. Thus, according to the zonal distribution given by Aufdenkampe et al. [8], tropical inland waters account for ∼60% of the global emission of CO_2_ from inland waters (0.45 PgC yr^−1^ for lakes and reservoirs, 0.39 PgC yr^−1^ for rivers and streams, and 1.12 PgC yr^−1^ for wetlands). It is clear that additional data are required to verify and re-evaluate more accurately the CO_2_ fluxes from tropical systems.

Pelagic particulate primary production (PP) of East African great lakes, as reviewed by Darchambeau et al. [15], ranges from ∼30 mmol m^−2^ d^−1^ for the most oligotrophic conditions (north basin of Lake Tanganyika) to ∼525 mmol m^−2^ d^−1^ for the most eutrophic conditions (Lake Victoria). The comparatively fewer data on bacterial production (BP), available only for Lake Tanganyika, suggest that PP and BP are seasonally closely coupled [16]. However, with an average pelagic BP of ∼25 mmol m^−2^ d^−1^ [16], the bacterial C demand would exceed the production of particulate organic C (POC) by phytoplankton in Lake Tanganyika. This has led to speculate about additional C supply to bacterioplankton, for instance, from dissolved organic C (DOC) exudation by phytoplankton [16].

Lake Kivu (2.50°S 1.59°S 29.37°E 28.83°E) is one of the East African great lakes (2370 km^2^ surface area, 550 km^3^ volume). It is a deep (maximum depth of 485 m) meromictic lake, with an oxic mixolimnion down to 70 m maximum, and a deep monolimnion rich in dissolved gases and nutrients [17], [18], [19]. Deep layers receive heat, salts, and CO_2_ from deep geothermal springs [19]. Seasonality of the physical and chemical vertical structure and biological activity in surface waters of Lake Kivu is driven by the oscillation between the dry season (June-September) and the rainy season (October-May), the former characterized by a deepening of the mixolimnion [20]. This seasonal mixing favours the input of dissolved nutrients and the development of diatoms, while, during the rest of the year, the phytoplankton assemblage is dominated by cyanobacteria, chrysophytes and cryptophytes [15], [21], [22]. Surface waters of Lake Kivu are oligotrophic, and, consequently, PP is at the lower end of the range for East African great lakes (on average ∼50 mmol m^−2^ d^−1^) [15].

Extremely high amounts of CO_2_ and methane (CH_4_) (300 km^3^ and 60 km^3^, respectively, at 0°C and 1 atm) [19] are dissolved in the deep layers of Lake Kivu. This is due to a steep density gradient at 260 m depth that leads to residence times in the order of 1000 yr in the deepest part of the lake [19], [23]. Stable isotope and radiocarbon data suggest that the CO_2_ is mainly geogenic [24]. While the risk of a limnic eruption is minimal [25], large scale industrial extraction of CH_4_ from the deep layers of Lake Kivu is planned [26], [27] which could affect the ecology and biogeochemical cycling of C of the lake and change for instance the emission of greenhouse gases such as CH_4_ and CO_2_. The net emission of CH_4_ to the atmosphere from Lake Kivu was quantified by Borges et al. [28], and was surprisingly low - among the lowest ever reported in lakes globally - considering the large amounts of CH_4_ stored in deep waters. Here, we report a data-set obtained during four surveys covering the seasonality of CO_2_ dynamics and fluxes, in conjunction with mass balances of C, and process rate measurements (PP and BP) in the epilimnion of Lake Kivu, with the aim of quantifying the exchange of CO_2_ with the atmosphere and determining the underlying drivers, in particular, the net metabolic status.

## Materials and Methods

Official permission was not required for sampling in locations where measurements were made and samples acquired. The field studies did not involve endangered or protected species. The full data-set is available in Table S1.

### 2.1 Field sampling and chemical analysis

**Figure 1:**
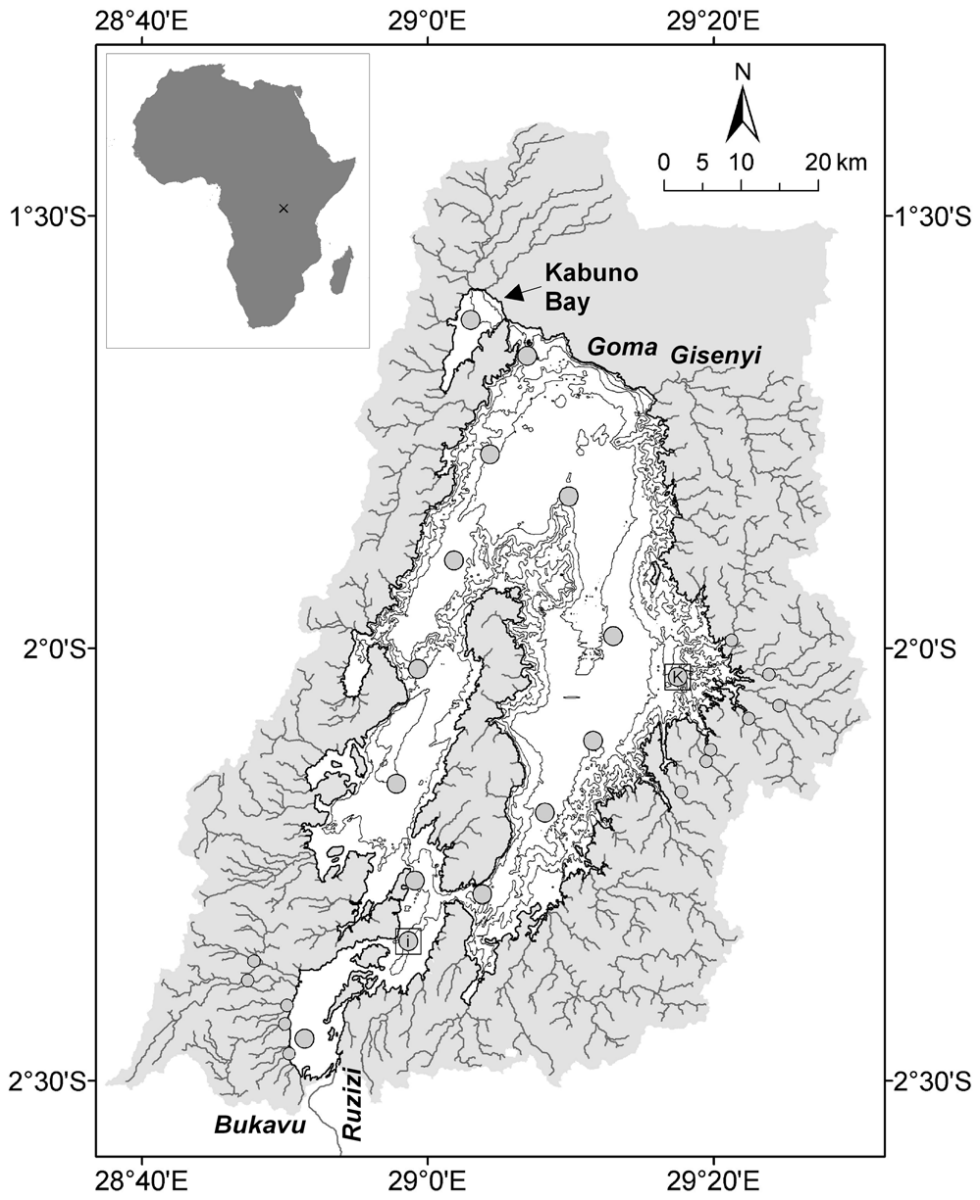
Map of Lake Kivu, showing bathymetry (isobaths at 100 m intervals), catchment area (shaded in grey), rivers, and sampling stations (small circles indicate the rivers). Primary production and bacterial production measurements were made at the stations identified with a square (I  =  Ishungu; K  =  Kibuye), adapted from [29].

In order to capture the seasonal variations of the studied quantities, four cruises were carried out in Lake Kivu on 15/03-29/03/2007 (mid rainy season), 28/08-10/09/2007 (late dry season), 21/06-03/07/2008 (early dry season), 21/04-05/05/2009 (late rainy season), and 19/10/10-27/10/10 (early rainy season) for a selection of variables. Sampling was carried out at 15 stations distributed over the whole lake and in Kabuno Bay, and at 12 rivers draining into Lake Kivu and representing the outflow of the lake (Ruzizi River, Fig. 1). The core of data presented hereafter was obtained in 2007–2009, while from the 2010 cruise only vertical DOC and POC data obtained at two stations are presented.

Vertical profiles of temperature, conductivity and oxygen were obtained with a Yellow Springs Instrument (YSI) 6600 V2 probe. Calibration of sensors was carried out prior to the cruises and regularly checked during the cruises. The conductivity cell was calibrated with a 1000 µS cm^−1^ (25°C) YSI standard. The oxygen membrane probe was calibrated with humidity saturated ambient air. Salinity was computed from specific conductivity according to Schmid and Wüest [29].

Sampling for the partial pressure of CO_2_ (pCO_2_) was carried out at 1 m depth. Measurements of pCO_2_ were carried out with a non-dispersive infra-red (NDIR) analyzer coupled to an equilibrator [30] through which water was pumped with a peristaltic pump (Masterflex E/S portable sampler). In-situ temperature and temperature at the outlet of the equilibrator were determined with Li-Cor 1000-15 probes. The NDIR analyzer (Li-Cor, Li-820) was calibrated with five gas standards: pure N_2_ and four CO_2_:N_2_ mixtures with a CO_2_ molar fraction of 363, 819, 3997 and 8170 ppm (Air Liquide Belgium).

For the determination of pH, CH_4_ concentrations, δ^13^C of dissolved inorganic C (DIC) (δ^13^C-DIC), and total alkalinity (TA), water was sampled with a 5 L Niskin bottle (Hydro-Bios). Samples were collected every 10 m from 10 to 60–80 m depending on the cruise and station, except for CH_4_ which was only sampled at 10 m. Additional samples for pH, δ^13^C_DIC_ and TA were collected at 5 m in Kabuno Bay. Water for CH_4_ analysis was collected in 50 ml glass serum bottles from the Niskin bottle with tubing, left to overflow, poisoned with 100 µl of a saturated HgCl_2_ solution, and sealed with butyl stoppers and aluminium caps. Water samples for the analysis of δ^13^C_DIC_ were taken from the same Niskin bottle by gently overfilling 12 ml glass Exetainer vials, poisoned with 20 µl of a saturated HgCl_2_ solution, and gas-tight capped. A water volume of 50 ml was filtered through a 0.2 µm pore size polyethersulfone (PES) syringe filters and was stored at ambient temperature in polyethylene bottles for the determination of TA. POC and DOC samples were obtained from surface waters in June 2008 and April 2009, and along a depth profile in October 2010. DOC was filtered on 0.2 µm PES syringe filters, stored at ambient temperature in 40 mL glass vials with polytetrafluoroethylene coated septa, and poisoned with 50 µL of H_3_PO_4_ (85%). POC was filtered on 0.7 µm pore 25 mm diameter Whatman GF/F glass fiber filters (pre-combusted 5 h at 500°C), stored dry. Sampling of river surface waters followed the same procedures outlined above (sampling depth ∼20 cm) with the addition of water sampling for total suspended matter (TSM). Samples for TSM were obtained by filtering 50–200 mL of water on pre-combusted pre-weighted 47 mm diameter GF/F glass fiber filters, stored dry.

Measurements of pH in water sampled from the Niskin bottle were carried out with a Metrohm (6.0253.100) combined electrode calibrated with US National Bureau of Standards (NBS) buffers of pH 4.002 (25°C) and pH 6.881 (25°C) prepared according to Frankignoulle and Borges [31]. Measurements of TA were carried out by open-cell titration with HCl 0.1 M according to Gran [32] on 50 ml water samples, and data were quality checked with Certified Reference Material acquired from Andrew Dickson (Scripps Institution of Oceanography, University of California, San Diego). DIC was computed from pH and TA measurements using the carbonic acid dissociation constants of Millero et al. [33]. For the analysis of δ^13^C-DIC, a He headspace was created in 12 ml glass vials, and ∼300 µl of H_3_PO_4_ (99%) was added to convert all DIC species to CO_2_. After overnight equilibration, part of the headspace was injected into the He stream of an elemental analyser – isotope ratio mass spectrometer (EA-IRMS) (ThermoFinnigan Flash1112 and ThermoFinnigan Delta+XL, or Thermo FlashEA/HT coupled to Thermo Delta V) for δ^13^C measurements. The obtained δ^13^C data were corrected for the isotopic equilibration between gaseous and dissolved CO_2_ using an algorithm similar to that presented by Miyajima et al. [34], and calibrated with LSVEC and NBS-19 certified standards or internal standards calibrated with the former. Concentrations of CH_4_ were determined by gas chromatography with flame ionization detection, as described by Borges et al. [28]. DOC and δ^13^C-DOC were measured with a customized Thermo HiperTOC coupled to a Delta+XL IRMS. POC and δ^13^C-POC from filters were determined on a Thermo EA-IRMS (various configurations, either Flash1112, FlashHT with Delta+XL or DeltaV Advantage). Quantification and calibration of δ^13^C data was performed with IAEA-C6 and acetanilide which was internally calibrated versus international standards.

PP and BP were measured at 2 stations: Kibuye (2.05°S 29.29°E) and Ishungu (2.34°S 28.98°E) (Fig. 1). PP was measured using the ^14^C method [35] as described by Darchambeau et al. [15] in a pooled sample prepared from discrete samples (2 L) spaced every 5 m in the mixed layer. The mixed layer depth (MLD) was determined from vertical profiles of temperature and oxygen. The ^14^C incubations were assumed to provide an estimate of net PP of the particulate phase (PNPP). Chlorophyll a (Chl-*a*) of the pooled sample was measured according to Descy et al. [36] by high-performance liquid chromatography analysis of extracts in 90% acetone from samples filtered on Macherey-Nägel GF5 (0.7 µm nominal pore size) filters (3–4 L). BP was estimated every 5 m in the mixed layer from tritiated thymidine (^3^H-Thy) incorporation rates [37]. Samples (20 mL) were incubated in duplicate for 2 h in the dark at in-situ temperature in the presence of ^3^H-Thy (∼80 Ci mmol) at saturating concentration (∼50 nmol L^−1^). After incubation, cold trichloroacetic acid (TCA) was added (final concentration 5%) and the samples were kept cold until filtration through a 0.2 µm pore-size cellulose nitrate membrane. Filters were preserved in the dark at −20°C. The radioactivity associated with the filters was estimated by liquid scintillation using a Beckman counter LS 6000. Cell production was calculated from the ^3^H-Thy incorporation rate using a conversion factor of 1.2×10^18^ cells produced per mole of ^3^H-Thy incorporated into cold TCA insoluble material. This conversion factor was determined experimentally in batch experiments in which the increase of bacterial abundance and ^3^H-Thy incorporation were followed simultaneously (data not shown) and was similar to the one used by Stenuite et al. [16] to calculate BP in Lake Tanganyika. Cellular production was multiplied by the average bacterial C content per cell (15 fgC cell^−1^) [16] to obtain BP data. Daily BP was estimated from the experimental values considering constant activity over 24 h, and expressed per unit area (mmol m^−2^ d^−1^), by integrating over the euphotic zone. Bacterial respiration (BR) rates were computed from BP using a bacterial growth efficiency computed from BP according to the model of Del Giorgio and Cole [38].

### 2.2 Bulk DIC mass balance model

TA and DIC mass balance models were constructed in order to determine the major processes controlling CO_2_ dynamics in surface waters, and to evaluate the net metabolic balance of the epilimnion (net autotrophy or net heterotrophy).

The TA mass balance was constructed assuming a steady-state, according to:
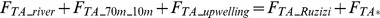
(1)where *F*_TA_river_ is the input of TA from rivers, *F*_TA_Ruzizi_ is the output of TA by the Ruzizi river, *F*_TA_70m_10m_ is the flux of TA from the monimolimnion to the mixolimnion by eddy diffusion, *F*_TA_upwelling_ is the flux of TA from the monimolimnion to the mixolimnion by upwelling, and *F*_TA*_ is the closing term.

*F*_TA_river_ was computed from discharge-weighted average TA in the 12 sampled rivers draining into Lake Kivu (TA_river_), and total freshwater discharge from rivers (Q_river_), according to:
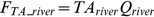
(2)


*F*_TA_Ruzizi_ was computed from TA measured in the Ruzizi River (TA_Ruzizi_), and the flow of the Ruzizi River (Q_Ruzizi_), according to:
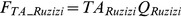
(3)


*F*_TA_70m_10m_ was computed from the gradient of TA across the pycnocline (δ_TA_70m_10m_/δz, where δz represents the depth interval) and the eddy diffusion coefficient (*E*) according to:
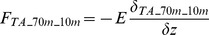
(4)


*F*_TA_upwelling_ was computed from the TA at 70 m (TA_70m_) and the upwelling flow (Q_upwelling_), according to:
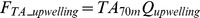
(5)


A DIC mass balance was constructed assuming a steady-state, according to:
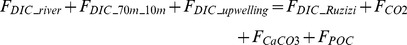
(6)where *F*_DIC_river_ is the input of DIC from rivers, *F*_DIC_Ruzizi_ is the output of DIC by the Ruzizi river, *F*_DIC_70m_10m_ is the flux of DIC from the monimolimnion to the mixolimnion by eddy diffusion, *F*_DIC_upwelling_ is the flux of DIC from the monimolimnion to the mixolimnion by upwelling, *F*_CO2_ is the exchange of CO_2_ with the atmosphere, *F*_CaCO3_ is the precipitation and subsequent export to depth of CaCO_3_, and *F*_POC_ is the closing term and represents the export of POC from surface to depth.

*F*_DIC_river_ was computed from discharge-weighted average DIC in the 12 sampled rivers draining into Lake Kivu (DIC_river_), and Q_river_, according to:
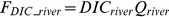
(7)


*F*_DIC_Ruzizi_ was computed from DIC measured in the Ruzizi River (DIC_Ruzizi_), and Q_Ruzizi_, according to:
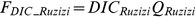
(8)


*F*_DIC_70m_10m_ was computed from the gradient of DIC across the metalimnion (δ_DIC_70m_10m_/δz) and *E* according to:
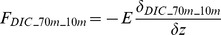
(9)


*F*_DIC_upwelling_ was computed from the DIC at 70 m (DIC_70m_) and Q_upwelling_, according to:
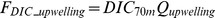
(10)


*F*_CO2_ was computed according to:
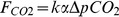
(11)where *k* is the gas transfer velocity, α is the CO_2_ solubility coefficient, and ΔpCO_2_ is the air-water gradient of pCO_2_ computed from water pCO_2_ (1 m depth) and an atmospheric pCO_2_ value ranging from ∼372 ppm to ∼376 ppm (depending on the cruise), corresponding to the monthly average at Mount Kenya (Kenya, 0.05°S 37.30°E) obtained from GLOBALVIEW-CO2 (Carbon Cycle Greenhouse Gases Group of the National Oceanic and Atmospheric Administration, Earth System Research Laboratory), and converted into wet air using the water vapour algorithm of Weiss and Price [39].

α was computed from temperature and salinity using the algorithm of Weiss [40], *k* was computed from wind speed using the parameterization of Cole and Caraco [41] and the Schmidt number of CO_2_ in fresh water according to the algorithm given by Wanninkhof [42]. Wind speed data were acquired with a Davis Instruments meteorological station in Bukavu (2.51°S 28.86°E). The wind speed data were adjusted to be representative of wind conditions over the lake by adding 2 m s^−1^ according to Thiery et al. [20]. *F*_CO2_ was computed with daily wind speed averages for a time period of one month centred on the date of the middle of each field cruise. Such an approach allows to account for the day-to-day variability of wind speed, and to provide *F*_CO2_ values that are seasonally representative.

*F*_CaCO3_ was computed according to:
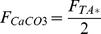
(12)


The average value of Q_river_ (76.1 m^3^ s^−1^) was given by Muvundja et al. [43], the average Q_Ruzizi_ for 2007–2009 (87.8 m^3^ s^−1^) measured at Ruzizi I Hydropower Plant was provided by the Société Nationale d'Electricité. A value of Q_upwelling_ of 42 m^3^ s^−1^ and a value of *E* of 0.06 cm^2^ s^−1^ were given by Schmid et al. [19].

### 2.3 δ^13^C-DIC mass balance model

The combination of the DIC and δ^13^C-DIC budget for the mixed layer allows to estimate independently the total DIC vertical input by upwelling and by eddy diffusion (*F*_DIC_upward_) and *F*_POC_ [44]. At steady-state, DIC and δ^13^C-DIC mass balances are given by the following equations:
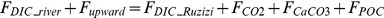
(13)




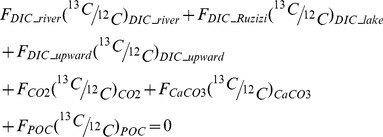
(14)

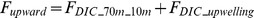
(15)


The (^13^C/^12^C) in the equation (14) represents the ^13^C to ^12^C ratio of net C fluxes, and can be expressed using the classical δ^13^C notation [45]. 10 out of the 12 different terms in equations (13) and (14) were measured or can be computed from measured variables, and then the two equations can be solved in order to estimate the *F*_DIC_upward_ and *F*_POC_ fluxes. *F*_DIC_river_, *F*_DIC_Ruzizi_, *F*_CaCO3_ and *F*_CO2_ were calculated as described above. (^13^C/^12^C)_DIC_river,_ (^13^C/^12^C)_DIC_lake_ and (^13^C/^12^C)_POC_ were measured during the 4 field surveys. (^13^C/^12^C)_CaCO3_ was computed from the measured δ^13^C_DIC_ in surface and the fractionation factor ε__CaCO3-HCO3_ of 0.88 ‰ [46].

The (^13^C/^12^C)_DIC_upward_ which represents the δ^13^C signature of the net upward DIC input, was estimated from the δ^13^C-DIC vertical gradient as follows: 
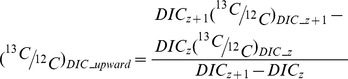
(16)where z is the depth, DIC_z_ and DIC_z+1_ are the DIC concentration at the depth z and z+1, (^13^C/^12^C)_DIC_z_ and (^13^C/^12^C)_DIC_z+1_ are the δ^13^C signature of DIC at the depth z and z+1.

The δ^13^C signature of the net flux of CO_2_ at the air-water interface was calculated from the δ^13^C signature of the different DIC species in surface water and the atmospheric CO_2_ (−8.0‰) according to: 
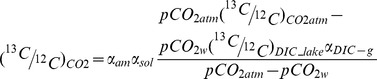
(17)where (^13^C/^12^C)_CO2atm_ and (^13^C/^12^C)_DIC_lake_ are the δ^13^C signature of atmospheric CO_2_ and lake surface DIC, respectively, α_am_ and α_sol_ are respectively the kinetic fractionation effect during CO_2_ gas transfer, and the equilibrium fractionation during CO_2_ dissolution measured by Zhang et al. [47] in distilled water. α_DIC-g_ is the equilibrium fractionation factor between aqueous DIC and gaseous CO_2_ and is defined by:
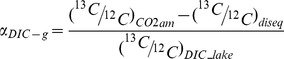
(18)where (^13^C/^12^C)_diseq_ is the air-water δ^13^C disequilibrium, that is the difference between the δ^13^C-DIC expected at equilibrium with atmosphere CO_2_ minus the measured δ^13^C-DIC in surface water of the lake.

### 2.4 Bulk DIC mixing models

A mixing model was developed to compute the theoretical evolution of TA, DIC, and pCO_2_ between March 2007 and September 2007, when the mixed layer deepened. The aim of this model is to compare theoretical evolution considering conservative mixing (no biology or other in/outputs) with observational data to infer the importance of certain processes. The model was computed by daily time steps assuming the conservative mixing (no biological activity) of surface waters with deep waters for TA and DIC. At each time step, pCO_2_ was calculated from TA, DIC, salinity and temperature, allowing the computation of *F*_CO2_ and correcting DIC for *F*_CO2_. The mixing model was also run without correcting DIC for *F*_CO2_. The MLD, salinity and temperature were interpolated linearly between March and September 2007.

At each time step, TA was computed according to:
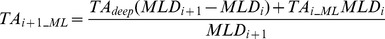
(19)where TA*_i_*__ML_ is TA in the mixed layer at time step *i*, TA*_i_*_+1_ML_ is TA in the mixed layer at time step *i*+1, MLD*_i_* is the MLD at time step *i*, MLD*_i_*_+1_ is the MLD at time step *i*+1, and TA_deep_ is TA in the deep waters.

At each time step, DIC corrected for *F*_CO2_ was computed according to:
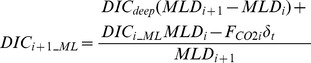
(20)where DIC*_i_*__ML_ is DIC in the mixed layer at time step *i*, DIC*_i_*_+1_ML_ is DIC in the mixed layer at time step *i*+1, *F*_CO2*i*_ is *F*_CO2_ at time step *i*, δt is the time interval between each time step (1 d), and DIC_deep_ is DIC in the deep waters.

At each time step, DIC not corrected for *F*_CO2_ was computed according to:
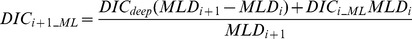
(21)


## Results

**Figure 2:**
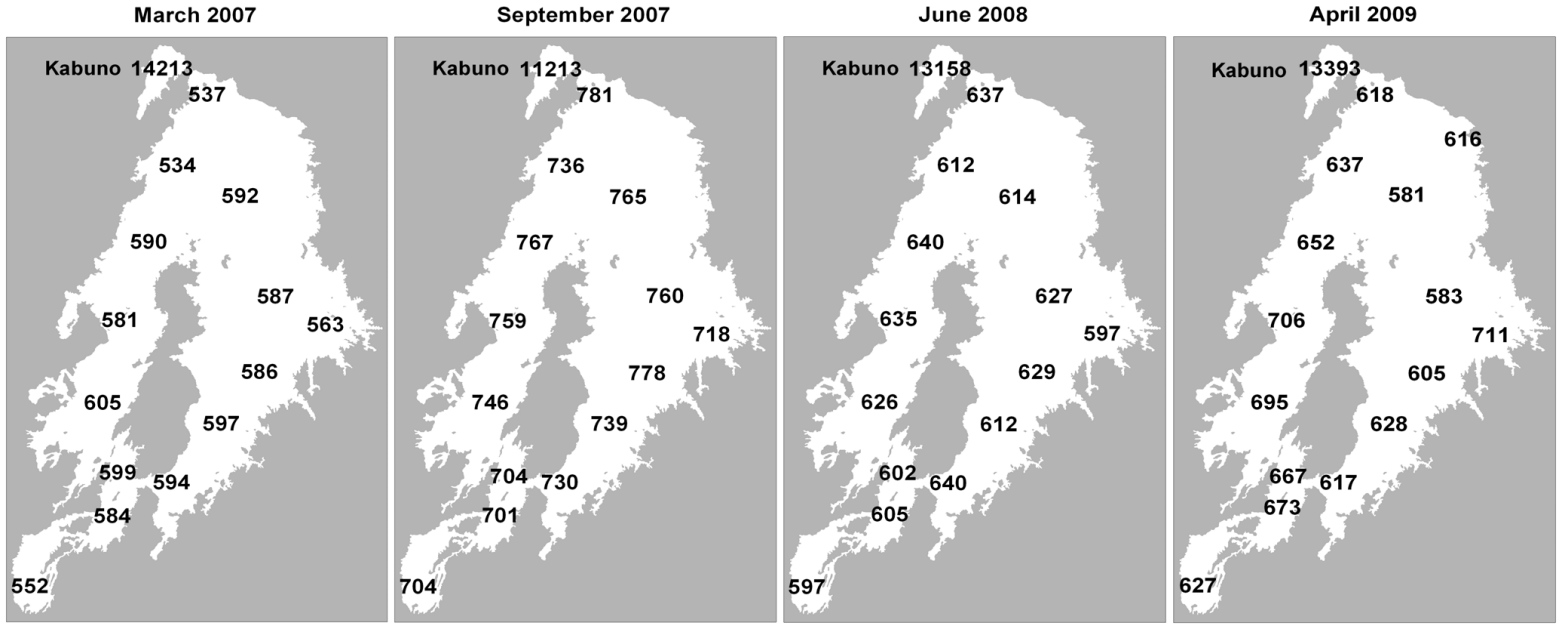
Spatial distribution of the partial pressure of CO_2_ (pCO_2_, ppm) in the surface waters of Lake Kivu (1 m depth) in March 2007, September 2007, June 2008 and April 2009.

In surface waters (1 m depth) of the main basin of Lake Kivu (excluding Kabuno Bay), pCO_2_ values were systematically above atmospheric equilibrium (∼372 ppm to ∼376 ppm depending on the cruise), and varied within narrow ranges of 534–605 ppm in March 2007, 701–781 ppm in September 2007, 597–640 ppm in June 2008, and 583–711 ppm in April 2009 (Fig. 2). The most prominent feature of the spatial variations was the much higher pCO_2_ values in Kabuno Bay, ranging between 11213 ppm and 14213 ppm (i.e., between 18 and 26 times higher than in the main basin). Wind speed showed little seasonal variability (ranging between 3.2 and 3.6 m s^−1^), hence, the seasonal variations of the CO_2_ emission rates followed those of ΔpCO_2_ with higher *F*_CO2_ values in September 2007 (14.2 mmol m^−2^ d^−1^) and lowest *F*_CO2_ in March 2007 (8.0 mmol m^−2^ d^−1^) in the main basin (Table 1). In Kabuno Bay, the *F*_CO2_ values ranged between 414.2 and 547.7 mmol m^−2^ d^−1^, and were on average ∼46 times higher than in the main basin.

**Table 1:** Average wind speed (m s^−1^), air-water gradient of the partial pressure of CO_2_ (ΔpCO_2_, ppm), and air-water CO_2_ flux (*F*_CO2_, mmol m^−2^ d^−1^) in the main basin of Lake Kivu and Kabuno Bay in March 2007, September 2007, June 2008, and April 2009.

		wind speed	ΔpCO_2_	*F*_CO2_
		(m s^−1^)	(ppm)	(mmol m^−2^ d^−1^)
March 2007				
	Main basin	3.3±0.4	207±22	8.0±1.3
	Kabuno Bay		13841	536.4±61.8
September 2007				
	Main basin	3.2±0.4	370±27	14.2±2.0
	Kabuno Bay		10841	547.7±38.7
June 2008				
	Main basin	3.6±0.2	245±15	10.5±1.0
	Kabuno Bay		12783	547.7±38.7
April 2009				
	Main basin	3.3±0.2	267±41	10.3±1.8
	Kabuno Bay		13016	504.5±36.7

**Figure 3:**
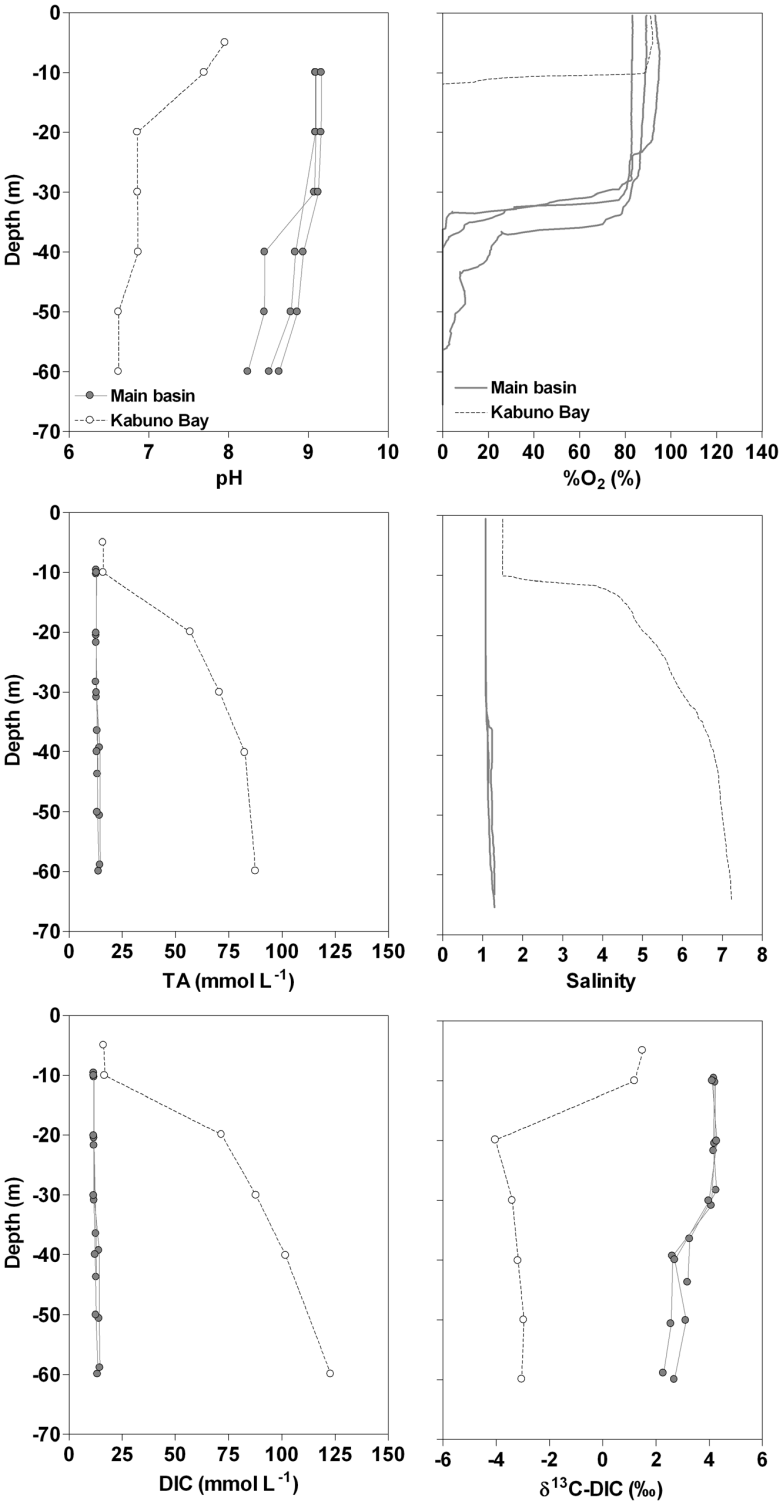
Vertical profiles in March 2007 of pH, oxygen saturation level (%O_2_, %), total alkalinity (TA, mmol L^−1^), salinity, dissolved inorganic carbon (DIC, mmol L^−1^), δ^13^C signature of DIC (δ^13^C-DIC, ‰) in Kabuno Bay and in the three northernmost stations in the main basin of Lake Kivu.

**Figure 4:**
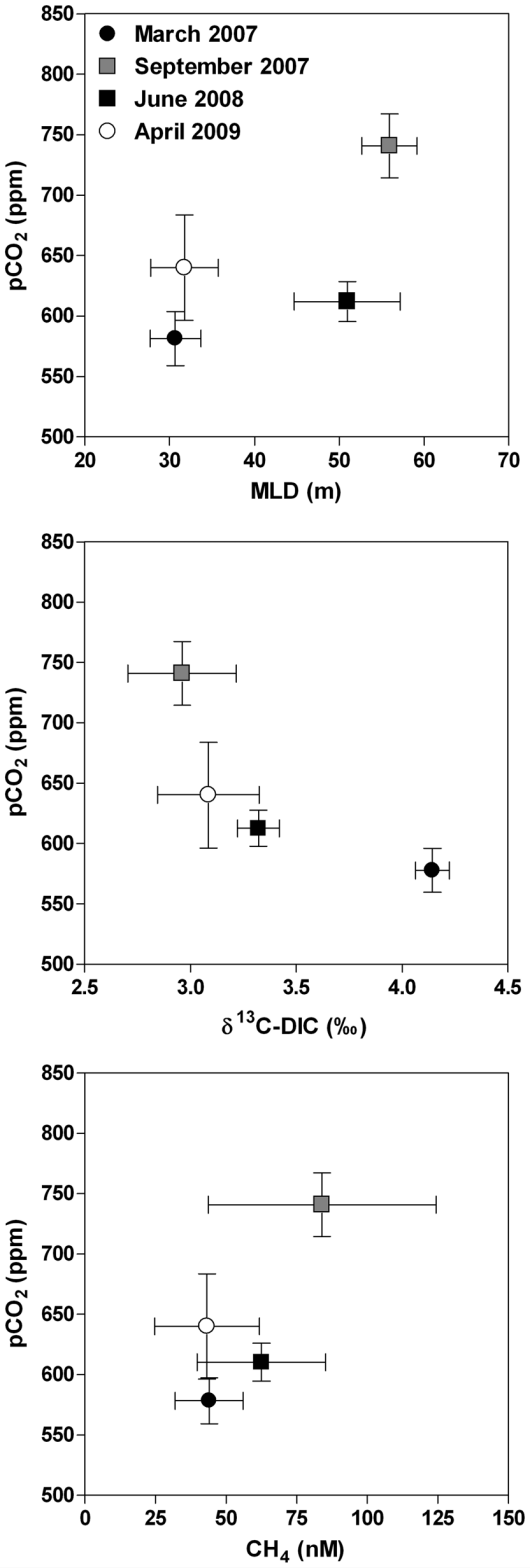
Average partial pressure of CO_2_ (pCO_2_, ppm) in the surface waters of the main basin of Lake Kivu (1 m depth) versus mixed layer depth (MLD, m), δ^13^C signature of dissolved inorganic carbon (DIC) (δ^13^C-DIC, ‰), and methane concentration (CH_4_, nmol L^−1^) in March 2007, September 2007, June 2008, and April 2009. Vertical and horizontal bars represent standard deviations.

Compared to the main basin, surface and deep waters of Kabuno Bay were characterized by higher salinity, DIC and TA values and by lower pH and δ^13^C-DIC values (Fig. 3). Comparison of DIC and TA profiles shows that the relative contribution of CO_2_ to DIC was more important in Kabuno Bay than in the main lake, since TA is mainly as HCO_3_^-^, and if the CO_2_ contribution to DIC is low, then DIC and TA should be numerically close. At 60 m depth, CO_2_ contributes ∼30% to DIC in Kabuno Bay, and only ∼1% in the main basin. Kabuno Bay was also characterized by a very stable chemocline (salinity, pH) and oxycline at ∼11 m irrespective of the sampling period [28]. In the main basin of Lake Kivu, the oxycline varied seasonally between ∼35 m in March and September 2007 and ∼60 m in June 2008 [28]. The deepening of the mixed layer and entrainment of deeper waters to the surface mixed layer was shown to be main driver of the seasonal variations of CH_4_ [28]. The positive correlations between pCO_2_ and CH_4_ and between pCO_2_ and the MLD also show that the mixing of deep and surface waters was a major driver of the seasonal variability of pCO_2_ (Fig. 4). This is also consistent with the negative relation between pCO_2_ and δ^13^C-DIC (Fig. 4), as DIC in deeper waters is more ^13^C-depleted than that in surface waters (Fig. 3).

**Figure 5:**
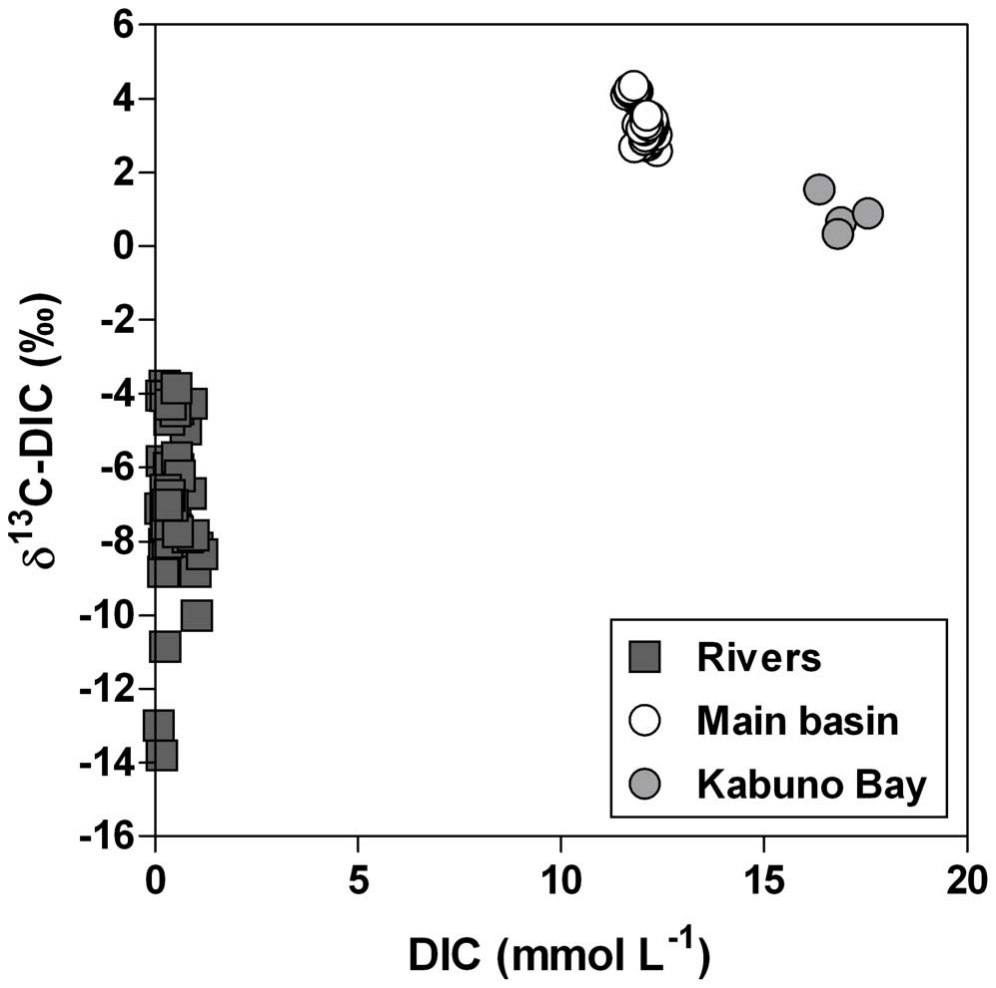
Relation between δ^13^C signature of dissolved inorganic carbon (DIC) (δ^13^C-DIC, ‰) and DIC concentration (mmol L^−1^), in the mixed layer of the main basin of Lake Kivu, Kabuno Bay, and various inflowing rivers, in March 2007, September 2007, June 2008, and April 2009.

**Figure 6:**
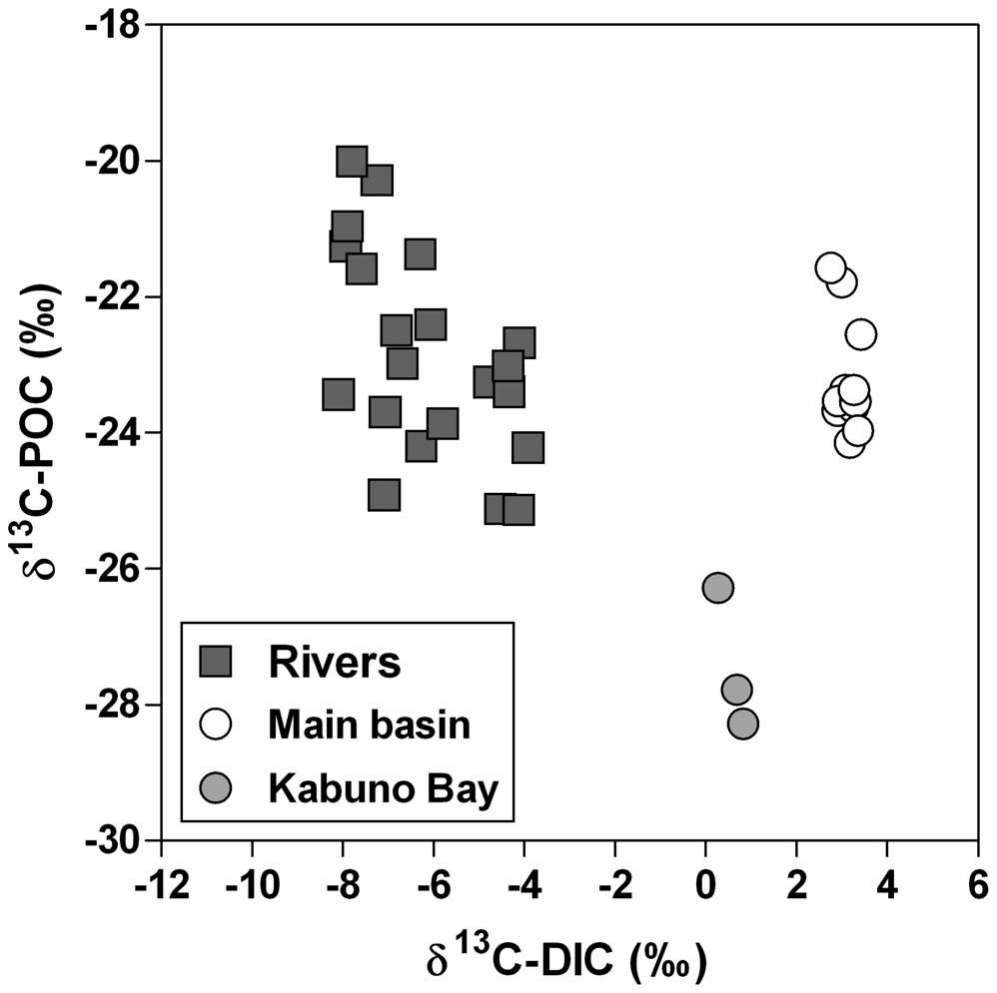
Relation between δ^13^C signature of particulate organic carbon (POC) (δ^13^C-POC, ‰) and δ^13^C signature of dissolved inorganic carbon (DIC) (δ^13^C-DIC, ‰), in the mixed layer of the main basin of Lake Kivu, Kabuno Bay and various inflowing rivers, in June 2008, April 2009, and October 2010.

**Figure 7:**
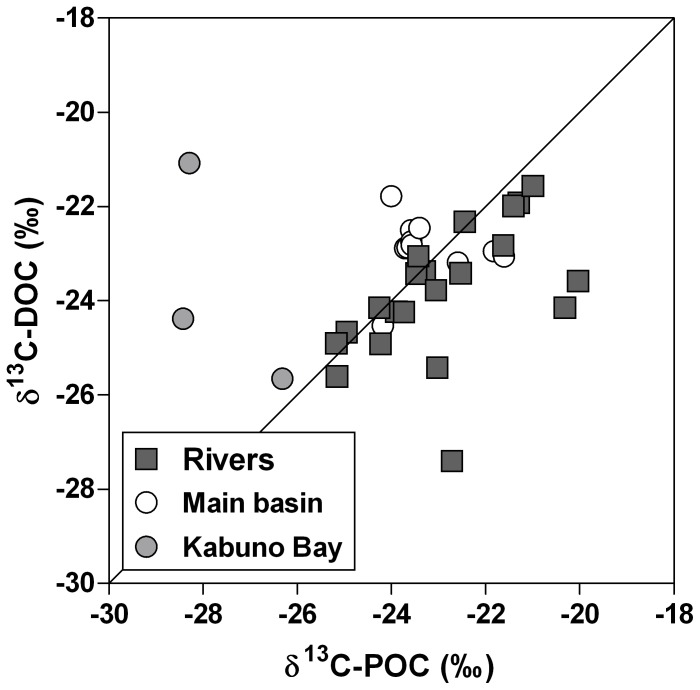
Relation between δ^13^C signature of dissolved organic carbon (DOC) (δ^13^C-DOC, ‰) and δ^13^C signature of particulate organic carbon (POC) (δ^13^C-POC, ‰), in the mixed layer of the main basin of Lake Kivu, Kabuno Bay and various inflowing rivers, in June 2008, April 2009, and October 2010. Solid line is the 1∶1 line.

**Figure 8:**
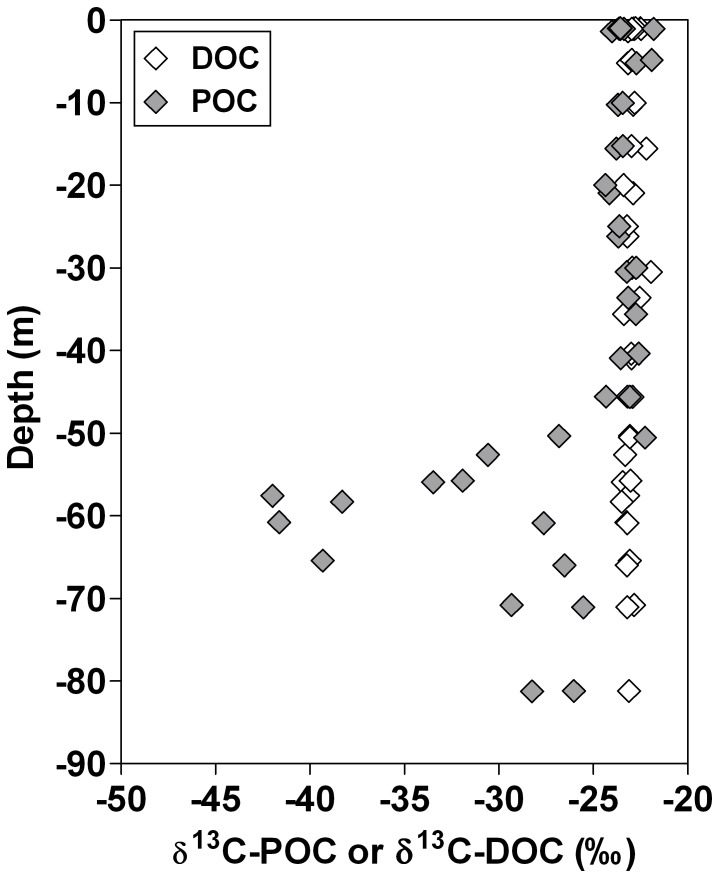
Vertical profiles of δ^13^C signature of dissolved organic carbon (DOC) (δ^13^C-DOC, ‰) and δ^13^C signature of particulate organic carbon (POC) (δ^13^C-POC, ‰) in the main basin of Lake Kivu in October 2010.

DIC concentrations in surface waters averaged 13.0 mmol L^−1^ and 16.9 mmol L^−1^ in the main basin of Lake Kivu and in Kabuno Bay, respectively, but were much lower in the inflowing rivers (on average ∼0.5 mmol L^−1^). The comparison with the lake values shows that the δ^13^C-DIC were always more negative in rivers (mean −7.0±2.1‰) than in the main basin (mean 3.4±0.5‰) and Kabuno Bay (mean 0.8±0.5‰) (Fig. 5). This difference suggests that the DIC in surface waters of Lake Kivu originates from a different source than that in the rivers. POC concentrations in surface waters of the main basin averaged 32 µmol L^−1^ in June 2008, 24 µmol L^−1^ in April 2009 and 42 µmol L^−1^ in October 2010. In the rivers, POC concentration was higher, 358 µmol L^−1^ in June 2008 and 499 µmol L^−1^ in April 2009. However, POC in the rivers never contributed more than 4.4% of TSM. δ^13^C-POC and δ^13^C-DIC signatures appeared uncoupled in rivers (Fig. 6), but a positive relationship between δ^13^C-DIC and δ^13^C-POC was found in the lake when combining the data from the main lake and Kabuno Bay (model I linear regression, *p*<0.001, *r* ^2^ = 0.71, n = 15). Furthermore, the δ^13^C-POC in the main basin and Kabuno Bay (mean −24.1±2.0‰, n = 15) was significantly lower than the δ^13^C-POC in rivers (mean = −22.9±1.5‰, n = 21) (*t*-test; p<0.05), but the δ^13^C-DOC in the main lake (mean −23.1±1.1‰, n = 15) did not differ from the δ^13^C-DOC in rivers (mean -23.9±1.4‰, n = 21) (Fig. 7) and was vertically uncoupled from δ^13^C-POC (Fig. 8).

**Figure 9:**
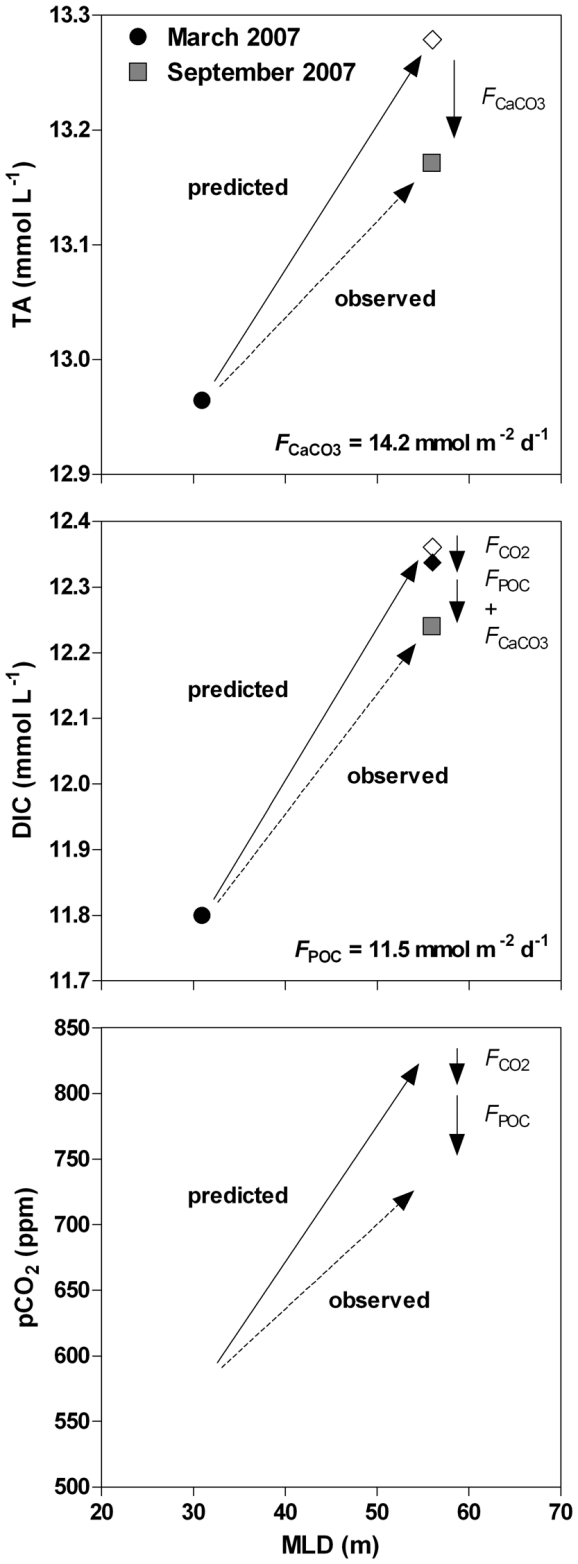
Observed data (circles and squares) and predicted values from a mixing model (diamonds) from March 2007 to September 2007 of total alkalinity (TA, mmol L^−1^), dissolved inorganic carbon (DIC, mmol L^−1^), and the partial pressure of CO_2_ (pCO_2_, ppm) as a function of mixed layer depth (MLD, m) in the main basin of Lake Kivu. *F*_CO2_  =  air-water CO_2_ flux; *F*_POC_  =  export of particulate organic carbon to depth; *F*_CaCO3_  =  export of CaCO_3_ to depth.

In order to test if vertical mixing was the only driver of seasonal variations of pCO_2_, we applied the mixing model to the March 2007 data in order to predict the evolution of TA, DIC, and pCO_2_ up to September 2007 and we compared the predicted values to the actual data obtained at that period (Fig. 9). The TA value predicted by the mixing model was higher than the observations in September 2007 by 108 µmol L^−1^. We assumed that the process removing TA was CaCO_3_ precipitation in the mixolimnion and subsequent export to depth (*F*_CaCO3_). In order to account for the difference between the mixing model prediction and the observations, *F*_CaCO3_ was estimated to be 14.2 mmol m^−2^ d^−1^ between March and September 2007.

The DIC value predicted by the mixing model was higher than the observations in September 2007 by 108 µmol L^−1^. The emission of CO_2_ to the atmosphere only accounted for 19% of the DIC removal. We assumed that the remaining DIC was removed by the combination of *F*_CaCO3_ and POC production in the epilimnion and export to depth (*F*_POC_) that was estimated to be 11.5 mmol m^−2^ d^−1^, using the *F*_CaCO3_ value estimated above from the TA data. The modeled pCO_2_ was above the observed pCO_2_ and the CO_2_ emission only accounted for 27% of the difference. This implies that the decrease of pCO_2_ was mainly related to *F*_POC_.

**Figure 10:**
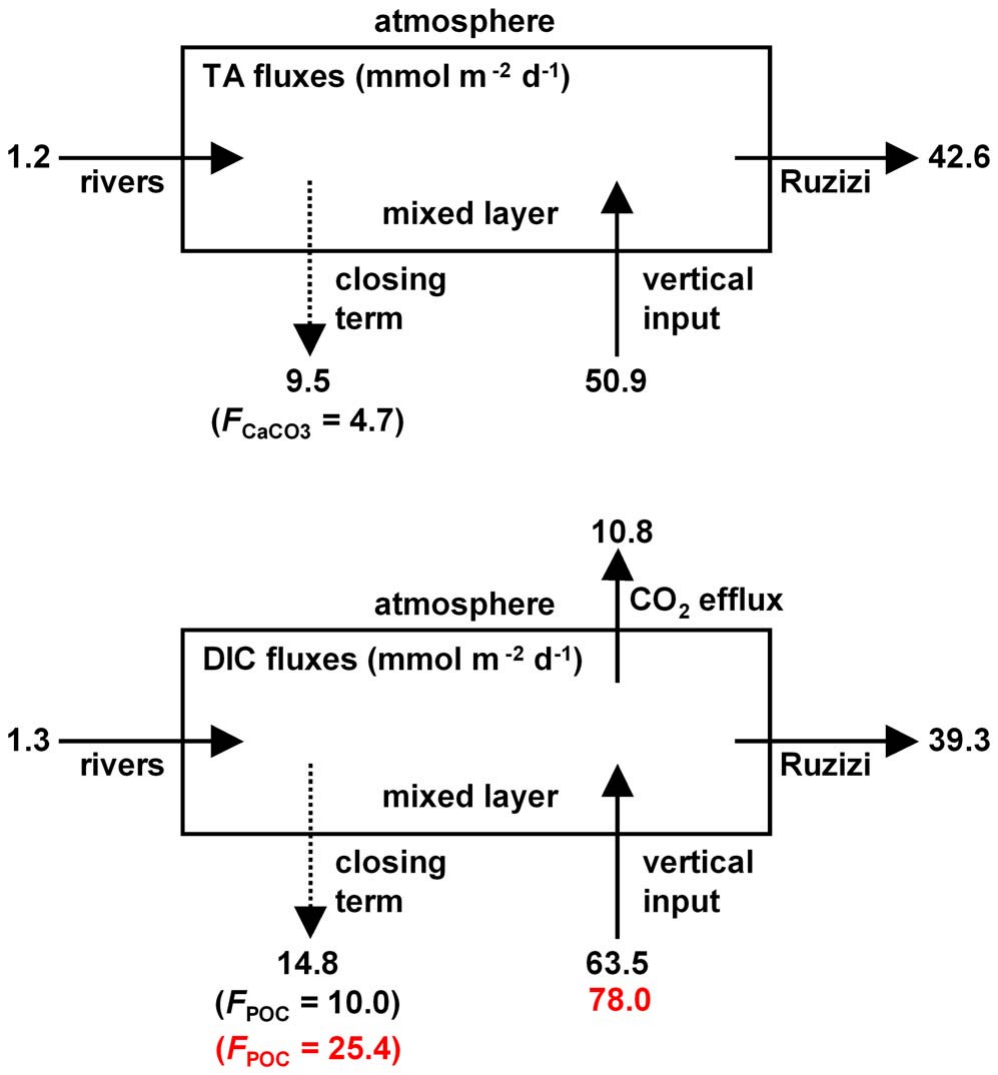
Average mass balance of total alkalinity (TA) and dissolved inorganic carbon (DIC) in the mixed layer of the main basin of Lake Kivu based on data collected in March 2007, September 2007, June 2008 and April 2009. *F*_POC_  =  export of particulate organic carbon to depth; *F*_CaCO3_  =  export of CaCO_3_ to depth. Numbers in black correspond to the mass balance based on bulk concentrations, and numbers in red correspond to the mass balance based on DIC stable isotopes. All fluxes are expressed in mmol m^−2^ d^−1^.

To further investigate the drivers of CO_2_ dynamics in Lake Kivu, we computed the TA and DIC whole-lake (bulk concentration) mass balances based on averages for the cruises (Fig. 10). The major flux of TA was the vertical input from deeper waters (50.9 mmol m^−2^ d^−1^) and the outflow by the Ruzizi (42.6 mmol m^−2^ d^−1^), which was higher than the inputs from rivers by one order of magnitude (1.2 mmol m^−2^ d^−1^). The closing term of the TA mass balance was 9.5 mmol m^−2^ d^−1^. We assume that this was related to *F*_CaCO3_ (4.7 mmol m^−2^ d^−1^). Similarly, the major fluxes of DIC were the vertical input (63.5 mmol m^−2^ d^−1^) and the outflow of the Ruzizi (39.3 mmol m^−2^ d^−1^), which were higher than the inputs from rivers by one order of magnitude (1.3 mmol m^−2^ d^−1^), and than the emission of CO_2_ to the atmosphere (10.8 mmol m^−2^ d^−1^). The closing term of the DIC mass balance was 14.8 mmol m^−2^ d^−1^. We assume that this was related to the sum of *F*_CaCO3_ and *F*_POC_, allowing to compute *F*_POC_ using the *F*_CaCO3_ values computed from the TA mass balance. The estimated *F*_POC_ values was 10.0 mmol m^−2^ d^−1^. The whole-lake DIC stable isotope mass balance provided a *F*_POC_ value of 25.4 mmol m^−2^ d^−1^, and vertical inputs of DIC of 78.0 mmol m^−2^ d^−1^.

Planktonic metabolic rates in the epilimnion (PNPP and BP) were measured during each cruise (Table 2). The PNPP values ranged from 14.2 to 49.7 mmol m^−2^ d^−1^, and were relatively similar in March 2007, September 2007 and June 2008, but distinctly lower in April 2009. The BP values ranged from 3.9 to 49.8 mmol m^−2^ d^−1^. This range encompasses the one reported for BP in the euphotic layer (∼40 m) of Lake Tanganyika (3.0 to 20.0 mmol m^−2^ d^−1^) [16]. The BR values estimated from BP ranged from 13.6 to 61.2 mmol C m^−2^ d^−1^. PNPP was markedly in excess of BR only in June 2008. In March 2007 and September 2007, BR was balanced by PNPP or slightly in excess of PNPP. In April 2009, BR was markedly in excess of PNPP.

**Table 2:** Photic depth (Z*e*, m), chlorophyll-a concentration in the mixed layer (Chl-*a*, mg m^-2^), particulate net primary production (PNPP, mmol m^−2^ d^−1^), bacterial production integrated over Z*e* (BP, mmol m^−2^ d^−1^), bacterial respiration (BR, mmol m^−2^ d^−1^), and percent of extracellular release (PER, %), at two stations in the main basin of Lake Kivu (Kibuye, Ishungu) in March 2007, September 2007, June 2008, and April 2009.

			*Ze*	Chl-*a*	PNPP	BP	BR	PNPP-BR	PER
			(m)	(mg m^−2^)	(mmol m^−2^ d^−1^)	(mmol m^−2^ d^−1^)	(mmol m^−2^ d^−1^)	(mmol m^−2^ d^−1^)	(%)
March 2007									
	15/03/2007	Kibuye	18	38.3	27.0	23.2	35.7	−8.7	54
	17/03/2007	Kibuye	20	48.4	42.5	25.8	39.9	2.6	33
	23/03/2007	Ishungu	17	36.1	49.7	40.5	49.6	0.1	32
September 2007									
	09/09/2007	Kibuye	19	56.4	42.9	35.9	47.9	−5.0	42
	12/09/2007	Kibuye	18	55.1	45.9	34.0	45.9	0.1	36
	04/09/2007	Ishungu	20	48.2	*n.d.*	16.0	29.9	*n.d.*	*n.d.*
June 2008									
	23/06/2008	Kibuye	24	42.8	46.0	7.7	21.6	24.4	3
	11/07/2008	Kibuye	20	37.8	42.0	11.1	24.3	17.7	17
	03/07/2008	Ishungu	19	28.1	40.7	3.9	13.6	27.1	*−4*
April 2009									
	04/05/2009	Kibuye	21	22.9	14.2	49.8	61.2	−47.0	82
	21/04/2009	Ishungu	24	39.3	24.5	43.5	58.9	−34.4	68

PNPP and BP were derived from experimental measurements. BR was computed from BP (see material and methods), and PER was computed from PNPP, BR, river inputs and vertical export of organic matter according to equation (24).

## Discussion

The amplitude of the seasonal variations of mean pCO_2_ across the main basin of Lake Kivu was 163 ppm (between 579±23 ppm on average in March 2007 and 742±28 ppm on average in September 2007). Such pCO_2_ seasonal amplitude is low compared to temperate and boreal lakes, where it is usually between ∼500 ppm [48] and>1000 ppm [49],[50],[51],[52],[53],[54], and even up to ∼10,000 ppm in small bog lakes [53]. The lower amplitude of seasonal variations of the pCO_2_ in Lake Kivu might be related to the tropical climate leading only to small surface water temperature seasonal variations (from 23.6°C in September 2007 to 24.6°C in March 2007 on average), and also for relatively modest variations in mixing (MLD changed from 20 m to 70 m). Hence, compared to temperate and boreal lakes, the seasonal variations of biological activity are less marked (due to relatively constant temperature and light, and modest changes in mixing), and also there is an absence of large episodic CO_2_ inputs to surface waters such as those occurring in temperate or boreal systems during lake overturns or of CO_2_ accumulation during ice covered periods.

The spatial variations of pCO_2_ in the main basin of Lake Kivu were also low. The coefficient of variation of pCO_2_ in surface waters of the main basin ranged for each cruise between 3% and 6%, below the range reported by Kelly et al. [54] in five large boreal lakes (range 5% to 40%). The relative horizontal homogeneity of pCO_2_ could be in part related to the absence of extensive shallow littoral zones, owing to the steep shores [18], and also due to very small influence of C inputs from rivers in the overall DIC budget (Fig. 10). The most prominent spatial feature in Lake Kivu was the much larger pCO_2_ values in surface waters of Kabuno Bay compared to the main basin. Furthermore, surface and deep waters of Kabuno Bay were characterized by higher salinity, DIC and TA values and by lower pH and δ^13^C-DIC values. These vertical patterns indicate that there is a much larger contribution of subaquatic springs to the whole water column including surface waters in Kabuno Bay than in the main basin of Lake Kivu relative to their respective volumes. This is related to the different geomorphology, since Kabuno Bay is shallower than the main basin (maximum depth of 110 m versus 485 m) and exchanges little water with the main basin (narrow connection ∼10 m deep). Also, Kabuno Bay is smaller (∼48 km^2^) than the main basin (∼2322 km^2^). Hence, there is a stronger fetch limitation of wind induced turbulence that also contributes to the stability of the vertical water column structure in Kabuno Bay irrespective of the season [28].

The overall average of pCO_2_ for the 4 cruises in the main basin of Lake Kivu (646 ppm) is lower than the average of 41 large lakes (>500 km^2^) of the world (850 ppm) [5], than the global average for freshwater lakes (1287 ppm) [12], than the average of tropical freshwater lakes (1804 ppm) [55], and than the average for tropical African freshwater lakes (2296 ppm) [49]. Lake Kivu corresponds to a saline lake according to the definition of Duarte et al. [56] (specific conductivity>1000 µS cm^−1^; salinity>0.68) that collectively have a global average pCO_2_ of 1900 ppm (derived from carbonic acid dissociation constants for freshwater) or 3040 ppm (derived from carbonic acid dissociation constants for seawater). Kabuno Bay, in contrast, was characterized by an exceptionally high average pCO_2_ value (12994 ppm) compared to other freshwater lakes, tropical (African) freshwater lakes, and saline lakes globally.

The average *F*_CO2_ of the 4 cruises in the main basin of Lake Kivu was 10.8 mmol m^−2^ d^−1^, which is lower than the global average for freshwater lakes of 16.0 mmol m^−2^ d^−1^ reported by Cole et al. [49], and the average for saline lakes ranging between 81 and 105 mmol m^−2^ d^−1^ reported by Duarte et al. [56]. The average *F*_CO2_ in Kabuno Bay (500.7 mmol m^−2^ d^−1^) is distinctly higher than the *F*_CO2_ global averages for freshwater and saline lakes. However, the average *F*_CO2_ in Kabuno Bay is equivalent to average of *F*_CO2_ value of alkaline volcanic lakes (458 mmol m^−2^ d^−1^) but lower than average of *F*_CO2_ of acid volcanic lakes (51183 mmol m^−2^ d^−1^) reported by Pérez et al. [57].

Cross system regional analyses show a general negative relationship between pCO_2_ and lake surface area [5], [54], [58], [59], [60] and a positive relationship between pCO_2_ and DOC [61] (and reference therein). The low pCO_2_ and *F*_CO2_ values in Lake Kivu are consistent with these general patterns, since this is a large (>2000 km^2^) and organic poor (DOC ∼0.2 mmol L^−1^) system. However, the low seasonal amplitude of pCO_2_ and relative horizontal homogeneity of pCO_2_ in Lake Kivu are not necessarily linked to its large size. Indeed, spatial and temporal variability of pCO_2_ within a single lake have been found to be no greater nor smaller in larger lakes than in smaller lakes, in cross system analyses in Northwest Ontario [54] and northern Québec [60].

Borges et al. [28] reported diffusive CH_4_ emissions of 0.04 mmol m^−2^ d^−1^ and 0.11 mmol m^−2^ d^−1^ for the main basin of Lake Kivu and Kabuno Bay, respectively. Using a global warming potential of 72 for a time horizon of 20 yr [62], the CH_4_ diffusive emissions in CO_2_ equivalents correspond to 0.26 mmol m^−2^ d^−1^ and 0.77 mmol m^−2^ d^−1^ for the main basin of Lake Kivu and Kabuno Bay, respectively, hence 41 to 650 times lower than the actual *F*_CO2_ values.

DIC concentrations in surface waters of the main basin of Lake Kivu and Kabuno Bay averaged 13.0 and 16.8 mmol L^−1^, respectively, and were well within the range of DIC reported for saline lakes by Duarte et al. [56], which range from 0.1 to 2140 mmol L^−1^, but are lower than the global average for saline lakes of 59.5 mmol L^−1^. DOC averaged in surface waters 0.15 mmol L^−1^ and 0.20 mmol L^−1^ in the main basin of Lake Kivu and in Kabuno Bay, respectively. Hence, DIC strongly dominated the dissolved C pool, with DIC:DOC ratios of 82 and 87 in the main basin of Lake Kivu and in Kabuno Bay, respectively. These DIC:DOC ratios are higher than those in 6 hard-water lakes of the northern Great Plains ranging from 3 to 6 [63], and higher than those in boreal lakes where DOC is the dominant form of the dissolved C pool, with DIC:DOC ratios ranging from 0.01 to 0.68 *e.g.* [64], [65], this range reflecting both seasonal changes [59] and differences in catchment characteristics [66]. Unlike the 6 hard-water lakes of the northern Great Plains, where the high DIC concentrations are due to river inputs [67], the high DIC concentrations in Lake Kivu were related to vertical inputs of DIC from deep waters that were on average 49 times larger than the DIC inputs from rivers (Fig. 10), as confirmed by δ^13^C-DIC values clearly more positive in the lake than in the rivers (Fig. 5). The difference in C stable isotope composition of POC between the lake and rivers indicates that these two pools of organic C do not share the same origin. In the small, turbid rivers flowing to Lake Kivu, we expect the POC and DOC pools to be derived from terrestrial inputs, as reflected by the low contribution of POC to TSM *e.g.* [68]. In contrast, the positive relationship between the δ^13^C-DIC and δ^13^C-POC in surface waters (Fig. 6) suggests that DIC is the main C source for POC in surface waters of Lake Kivu, implying that the whole microbial food web could be supported by autochthonous organic C. However, the δ^13^C data indicate a surprising difference between the origin of DOC and POC in the lake (Figs. 7, 8). The δ^13^C-POC signatures were constant from the surface to the oxic-anoxic interface, then showed a local and abrupt excursion to values as low as −40‰, reflecting the incorporation of a ^13^C-depleted source in the POC (Fig. 8). Indeed, while the large pool of DIC is the main C source for POC in surface waters, it appears that CH_4_ with a δ^13^C signature of approximately −60‰ (own data not shown) contributes significantly to C fixation at the oxic-anoxic interface, as also shown in Lake Lugano [69]. In contrast, the δ^13^C signature of the DOC pool in the mixolimnion showed little seasonal and spatial variations and appeared to be uncoupled from the POC pool (Figs. 7, 8). Heterotrophic bacteria quickly mineralized the labile autochthonous DOC that reflects the δ^13^C signature of POC, produced by cell lysis, grazing, or phytoplankton excretion [70]. Hence, standing stocks of autochthonous DOC are small, and older refractory compounds constitute the major part of the DOC pool.

The *F*_CaCO3_ value computed from the whole-lake TA budget was 4.7 mmol m^−2^ d^−1^, higher than the total inorganic C (TIC) annual average fluxes in sediment traps ranging from 0.3 mmol m^−2^ d^−1^ (at 50 m depth) to 0.5 mmol m^−2^ d^−1^ (at 130 m depth) reported by Pasche et al. [71] at Ishungu. The maximum individual monthly TIC flux from sediment traps at Ishungu reported by Pasche et al. [71] was 4.0 mmol m^−2^ d^−1^. However, the *F*_CaCO3_ value was closer to the TIC deposition fluxes in the top cm of sediment cores ranging from 1.4 mmol m^−2^ d^−1^ (at Ishungu) to 4.8 mmol m^−2^ d^−1^ (at Gisenyi) also reported by Pasche et al. [71]. The *F*_POC_ computed from the DIC whole-lake budget was 10.0 mmol m^−2^ d^−1^ close to the total organic C (TOC) average fluxes in sediment traps ranging from 8.7 mmol m^−2^ d^−1^ (at 50 m depth) to 9.8 mmol m^−2^ d^−1^ (at 172 m depth) reported by Pasche et al. [71] at Ishungu.

Due to thermodynamic equilibria of the dissolved carbonate system, CaCO_3_ precipitation leads to a shift from the HCO_3_^-^ to the CO_2_ pool according to:
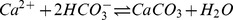
(22)


However, CaCO_3_ precipitation has frequently been reported in lakes as biologically mediated by primary producers [50], [72], [73], whereby the CO_2_ produced by the precipitation of CaCO_3_ is fixed into organic matter by photosynthesis and does not accumulate in the water [50], [74].

Precipitation and preservation of CaCO_3_ in lakes are not considered in global compilations of C fluxes in lakes, that focus exclusively on organic C and CO_2_ fluxes *e.g.* [4], [7]. However, in Lake Kivu, *F*_CaCO3_ was found to be a major flux term in the C budgets, 3.6 times larger than the DIC inputs from rivers, and comparable to the emission of CO_2_ to the atmosphere and *F*_POC_ (∼2 times lower). The *F*_POC_:*F*_CaCO3_ ratio in the main basin of Lake Kivu was 2.1, which is consistent with the values reported in 6 hard-water lakes of the northern Great Plains, ranging from 1.0 to 4.0 [63], and with the values in Lake Malawi ranging from 0.2 to 7.3 (on average 2.5) [75]. As a comparison, the average *F*_POC_:*F*_CaCO3_ in the ocean (so called rain ratio) has been estimated from models of varying complexity to be 4.0 [76], 3.5 to 7.5 [77], and 11.0 [78].

The export ratio (ER) is the fraction of PP that is exported from surface waters to depth and is an important metric of the net metabolism and overall C fluxes in aquatic systems. We computed ER as defined by Baines et al. [79], according to:
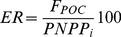
(23)where *F*_POC_ is derived from the DIC mass balance (Fig. 10), and PNPP*_i_* is the average PNPP for a given cruise *i* measured by incubations (Table 2).

ER was 25%, 23%, 23%, and 52% in March 2007, September 2007, June 2008, and April 2009, respectively. These values are consistent with the fact that the ER in lakes is negatively related to lake primary production based on the analysis of Baines et al. [79]. These authors reported ER values as high as 50% for oligotrophic lakes such as Lake Kivu.

The general agreement between the *F*_CaCO3_ computed from the TA budget and the TIC deposition fluxes derived from sediment cores reported by Pasche et al. [71], and the *F*_POC_ computed from the DIC budget and TOC average fluxes in sediment traps reported by Pasche et al. [71], give confidence on the overall robustness of the TA and DIC whole-lake budget we computed. Also, the *F*_CaCO3_ and *F*_POC_ values computed from whole-lake budget are consistent with those derived independently from a mixing model based on the March and September 2007 data (Fig. 9). The whole-lake DIC stable isotope mass balance budgets give *F*_POC_ and upward DIC inputs estimates that are of same order of magnitude as those derived from whole-lake bulk DIC mass balance budget. The difference in the two approaches is that in the DIC stable isotope mass balance the upward DIC inputs were computed from vertical distributions of DIC and δ^13^C-DIC while in the bulk DIC mass balance they are computed from the *E* and Q_upwelling_ values from the model of Schmid et al. [19] and the DIC vertical distribution. This can explain the mismatch between both approaches in the upward DIC input estimates (difference of 23%) that propagated into a relatively larger mismatch in the *F*_POC_ estimates (difference of 61%) computed as a closing term in both approaches.

Based on the POC and DOC data acquired during the June 2008 and April 2009 cruises in 12 rivers flowing into Lake Kivu (Fig. 1), we computed an overall TOC input from rivers of 0.7 mmol m^−2^ d^−1^ and 3.3 mmol m^−2^ d^−1^, respectively. The *F*_POC_ was 10 mmol m^−2^ d^−1^, implying a net organic C production in the epilimnion (net autotrophic community metabolic status). This would mean that the fraction of PNPP that does not sediment out of the epilimnion cannot meet BR, and that BR must then rely on other organic C sources. We assume these other organic C sources to be dissolved primary production (DPP), that was estimated assuming steady-state, according to:
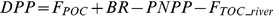
(24)where *F*_TOC_river_ is the input of TOC from rivers that was computed from the discharge weighted average TOC concentrations from the June 2008 and April 2009 cruises.

The percent of extracellular release (PER) allows to determine the relative importance of DPP in overall C flows in an aquatic system. PER as defined by Baines and Pace [80] was computed according:
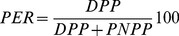
(25)


In June 2008, for two stations, the sum of organic C inputs (PNPP + *F*_TOC_river_) exceeded the sum of organic C outputs (*F*_POC_ + BR), leading to negative DPP and PER estimates. If we exclude these values, PER estimates ranged from 3% to 80% (Table 2) encompassing the range reported by Baines and Pace [80] for freshwater lakes from ∼0% to ∼75%. PNPP in April 2009 was distinctly lower than during the other cruises, leading to high PER estimates. The average PER for all cruises was 32%, and if the April 2009 data are excluded, the average PER was 19%. During the April 2009 field survey, we carried 6 h incubations using the ^14^C incorporation method [81] in light controlled (200 µE m^−2^ s^−1^) conditions, allowing to measure PNPP and to compute DPP using the model of Moran et al. [82]. Experimentally-derived PER estimates were 57% at Ishungu, 62% at Kibuye, and 50% in Kabuno Bay [70]. These experimentally determined PER values are within the range of those determined from the mass balance (3% to 80%) and above the average for all cruises (32%). This confirms that a substantial part of the BR is subsidised by DPP, that part of the PNPP is available for export to depth, and consequently that the epilimnion of Lake Kivu is net autotrophic, although a source of CO_2_ to the atmosphere.

## Conclusions

Surface waters of 93% of the lakes in the compilation of Sobek et al. [12] were over-saturated in CO_2_ with respect to the atmospheric equilibrium. Hence, the overwhelming majority of lakes globally act as a CO_2_ source to the atmosphere. These emissions to the atmosphere have been frequently explained by the net heterotrophic nature of lakes sustained by terrestrial organic C inputs mainly as DOC [1], [6], [7], [12], [49], [83], [84], [85], [86], [87]. While this paradigm undoubtedly holds true for boreal humic lakes, several exceptions have been put forward in the literature. For instance, Balmer and Downing [88] showed that the majority (60%) of eutrophic agriculturally impacted lakes are net autrotrophic and CO_2_ sinks. Karim et al. [89] showed that surface waters of very large lakes such as the Laurentian Great Lakes are at equilibrium with atmospheric CO_2_ and O_2_. This is consistent with the negative relationship between pCO_2_ and lake size reported in several regional analyses [5], [54], [58], [59], [60], and with the positive relationship between pCO_2_ and catchment area: lake area reported for Northern Wisconsin lakes [90]. Also, in some lakes among which hard-water lakes, the magnitude of CO_2_ emissions to the atmosphere seems to depend mainly on hydrological inputs of DIC from rivers and streams [67], [91], [92], [93] or ground-water [52], [94], [95], rather than on lake metabolism. Some of these hard-water lakes were actually found to be net autotrophic, despite acting as a source of CO_2_ to the atmosphere [67], [91], [93].

Here, we demonstrate that Lake Kivu represents an example of a large, oligotrophic, tropical lake acting as a source of CO_2_ to the atmosphere, despite having a net autotrophic epilimnion. The river inputs of TOC were modest, and were on average 11 times lower than the export of POC to depth. This is probably related to the very low ratio of catchment surface area: lake surface area (5100∶2370 km^2^∶km^2^) that is among the lowest in lakes globally [96]. We showed that BR was in part subsidized by DPP, based on mass balance considerations and incubations. Since the epilimnion of Lake Kivu is net autotrophic, the CO_2_ emission to the atmosphere must be sustained by DIC inputs. The river DIC inputs are also low owing to very low ratio of catchment surface area: lake surface area, and cannot sustain the CO_2_ emission to the atmosphere unlike the hard water lakes studied by Finlay et al. [67] and Stets et al. [91]. In Lake Kivu, the CO_2_ emission is sustained by DIC inputs from depth, and this DIC is mainly geogenic [24] and originates from deep geothermal springs [19].

Carbonate chemistry in surface waters of Lake Kivu is unique from other points of view. The dissolved C pool is largely dominated by DIC, with DIC:DOC ratios distinctly higher than in hard-water lakes and humic lakes. The high DIC content in surface water results in CaCO_3_ over-saturation, in turn leading to CaCO_3_ precipitation and export to depth. This flux was found to be significant, being 4 times larger than the river inputs of DIC and of similar magnitude than the CO_2_ emission to the atmosphere.

## Supporting Information

Table S1 Data-set of depth (m), water temperature (°C), specific conductivity at 25°C (µS cm^−1^), oxygen saturation level (%O_2_, %), δ^13^C signature of dissolved inorganic carbon (DIC) (δ^13^C-DIC, ‰), total alkalinity (TA, mmol L^−1^), pH, partial pressure of CO_2_ (pCO_2_, ppm), dissolved methane concentration (CH_4_, nmol L^−1^), particulate organic carbon (POC, mg L^−1^), δ^13^C signature of POC (δ^13^C-POC, ‰), dissolved organic carbon (DOC, mg L^−1^), δ^13^C signature of DOC (δ^13^C-DOC, ‰), total suspended matter (TSM, mg L^−1^) in Lake Kivu and 12 rivers flowing into Lake Kivu, in March 2007, September 2007, June 2008, April 2009 and October 2010.
(XLS)
